# Editorial: A Hand at Work: Effects of Aging

**DOI:** 10.3389/fnagi.2016.00141

**Published:** 2016-06-14

**Authors:** Pranav J. Parikh, Kelly J. Cole

**Affiliations:** ^1^Department of Health and Human Performance, Center for Neuromotor and Biomechanics Research, University of HoustonHouston, TX, USA; ^2^School of Biological and Health Systems Engineering, Arizona State UniversityTempe, AZ, USA; ^3^Department of Health and Human Physiology, University of IowaIowa City, IA, USA

**Keywords:** GRASP, cortex, sensorimotor integration, interlimb, variability

Most older adults self-report disability for daily activities using their hands. Scientists have attempted to characterize specific behavioral markers of sensorimotor dysfunction, identify the changes in central and peripheral nervous systems, and establish a link between behavioral and neurophysiological alterations with aging. Neuroimaging studies have reported associations between changes in the brain and performance on cognitive and motor tasks in older adults (Sailer et al., 2000; Cabeza et al., 2002; Park et al., 2004; Park and Reuter-Lorenz, 2009; Carp et al., 2011; Bernard and Seidler, 2012; Langan et al.). For example, among older adults greater brain activation levels associated with better motor performance, which suggest over-activation of the brain (compared to young adults) serves to compensate for age-related deterioration of brain function (Mattay et al., 2002; Rossi et al., 2004; Wu and Hallett, 2005; Heuninckx et al., 2008). The over-activation may hinder performance due to the difficulty in selectively recruiting neural networks, which instead is interpreted to reflect reduced brain circuit differentiation (Peinemann et al., 2001; Riecker et al., 2006; Dennis and Cabeza, 2011; Seidler et al., 2011; Bernard and Seidler, 2012; Langan et al.). As certain age-related neurophysiological changes may be beneficial, it is important to understand how age-related changes in the nervous system correlate with behavioral outcomes. These studies further depend on identification of behavior and neurophysiological measures that are sensitive to aging.

Studies published as a part of this research topic used innovative approaches to assess behavioral and neurophysiological deficits in older adults. These approaches ranged from the utilization of behavioral paradigms to neuroimaging tools that focused on unimanual motor tasks (Bonstrup et al.; de Bruin et al.; Kimura et al.; Ko et al.; Lawrence et al.; Tracy et al.; Yu et al.) or bimanual tasks (Bhakuni and Mutha; Boisgontier and Swinnen; Boisgontieret al. Chen et al.; Hoff et al.).

Bimanual behavioral paradigms allow characterization of complex interaction between two sides of the brain during performance of a motor task. Boisgontier et al. used a bimanual joint position matching task that required reliance on proprioceptive information from both limbs in order to match the position of one limb to another. The authors found that directing visual attention to the active limb impaired performance in both young and older adults, but more so in older adults (Boisgontier et al.). This finding was suggested to arise from older adults' inability to handle additional attention load, in the presence of an underlying age-related increased cost of processing proprioceptive signals. Further, older adults were impaired when the target limb was placed farther away, compared to near targets (Boisgontier and Swinnen). For the farthest targets older adults most likely experienced an increase in proprioceptive signal processing load and/or an increase in noise in the joint proprioceptor firing rate. The authors suggested that these factors might have saturated the reserve for proprioceptive processing in those older adults who were dependent on compensatory mechanisms for task performance, thus impairing performance on the joint matching task.

The interaction between age and interlimb coordination during skill learning was investigated using a bimanual serial reaction time task (Bhakuni and Mutha; Hoff et al.). Older adults were found to match their younger counterparts in the ability to learn a serial reaction time task (Bhakuni and Mutha; Hoff et al.). However, Ragert and colleagues noted that older adults demonstrated reduced sequence specific learning as compared with younger individuals. Importantly, older adults showed altered interlimb coordination, measured as the time cost related to hand switch (i.e., switch cost), when compared with young adults during performance of the bimanual task (Bhakuni and Mutha; Hoff et al.). While Bhakuni and Mutha reported no change in the hand switch cost with skill learning in both older and young individuals, Hoff et al. found that the hand switch cost in older adults declined to levels that matched younger individuals. In a study of animals, Chen et al. found that increased neonatal iron supplementation resulted in the depletion of striatal dopamine and reduction of motor performance on rotarod and open field tests in aging, but not in young, rats.

Other studies in this special section quantified sensorimotor integration processes using unimanual tasks in elderly individuals. Specifically, Valero-Cuevas and colleagues used a novel behavioral paradigm, the Strength-Dexterity (SD) test, which provides information about the sensorimotor integration processes through quantification of the dynamic control of fingertip forces. They found that sensorimotor ability assessed using the SD test significantly contributes to hand function in the aging population, in addition to strength and coordinated upper extremity function (Lawrence et al.). Poor unimanual hand function in older adults has also been shown to result from increased variability in motor output, e.g., forces exerted on an object (Enoka et al., 2003; Christou, 2011). The age-related increase in force variability during isometric force production task is noted specifically when the task is performed under the influence of visual feedback of the applied force and the target force. No or minimal age-related difference was observed for tasks performed in the absence of visual feedback. However, the age-related and vision-dependent increased force variability was mainly reported for large muscle groups (Tracy, 2007; Tracy et al., 2007; Welsh et al., 2007; Paxton et al., 2015). Here, Tracy et al. showed age-related increase in force variability under high-gain visual feedback for an intrinsic muscle of the hand. Overall, the finding of increased force variability during an isometric task across small and large muscle groups in old age mainly when vision is made available suggests impaired processing of visuomotor information during isometric task with aging. In contrast, older adults with a co-morbid condition may demonstrate lower variability in the motor output suggestive of their reduced flexibility and adaptability to unpredictable environmental conditions. Valero-Cuevas and colleagues found that older adults with Parkinson's disease (PD) have lower force variability during a dynamic stability (i.e., the SD) test as compared with healthy older adults (Ko et al.). Importantly, the authors found that the reduced force variability correlated with greater severity of motor impairment assessed using UPDRS motor scores. Overall, these studies suggest that it is crucial to understand the relationship between motor output variability and hand function with aging when associated with other health-related problems. Yu et al. reported that older women as compared with older men have a higher prevalence of severe stroke, hypertension, obesity, and hyperlipidemia. De Bruin et al. developed a novel task to assess the effects of aging on visuospatial abilities, but failed to find any age-related decrement. Lastly, Kimura et al. studied the effects of aging on performing speedy corrective movements when the target jumps during the reach. The authors inferred that older adults potentially have intact reflexive, but impaired volitional, corrective responses to target jumps during the reach.

In addition to behavioral metrics, metrics that quantify structural and functional changes in the brain will be useful to understand the effects of aging on the sensorimotor system. Bonstrup et al. investigated a cortical signature for sustained inhibition of finger movement, e.g., an increase in alpha electroencephalography (EEG) power, in young and older adults. The authors found a significant increase in alpha power over sensorimotor cortices during sustained inhibition of a learned complex finger movement sequence in young adults, but not in older adults. This finding suggests that older adults have impaired inhibitory mechanisms necessary for the temporal control of movement, and that the EEG alpha power can be a sensitive tool to detect any age-related deterioration of inhibitory neural mechanisms.

The studies compiled within this special issue are intended to promote discussion on the sensorimotor deficits that elderly individuals face and will encourage further behavioral and neurophysiological studies to investigate the specific neural mechanisms.

## Author contributions

PP drafted manuscript, PP and KC edited and revised manuscript, PP and KC approved the final version of manuscript.

### Conflict of interest statement

The authors declare that the research was conducted in the absence of any commercial or financial relationships that could be construed as a potential conflict of interest.
